# Impact of timing of trastuzumab initiation on long-term outcome of patients with early-stage HER2-positive breast cancer: the “one thousand HER2 patients” project

**DOI:** 10.1038/s41416-018-0114-x

**Published:** 2018-05-18

**Authors:** Giuseppe Gullo, Naomi Walsh, David Fennelly, Reetesh Bose, Janice Walshe, Dimitrios Tryfonopoulos, Kate O’Mahony, Lisa Hammond, Nuno Silva, Deirdre McDonnell, Josephine Ballot, Cecily Quinn, Enda W. McDermott, Denis Evoy, Ruth Prichard, James Geraghty, John Amstrong, John Crown

**Affiliations:** 1 Department of Medical Oncology, St Vincent’s University Hospital, Dublin, Ireland; 2 School of Medicine, University College Dublin (UCD), Dublin, Ireland; 3 National Institute for Cellular Biotechnology, Dublin City University (DCU), Dublin, Ireland; 4 Department of Medical Oncology, Agios Savvas Hospital, Athens, Greece; 5 Aseptic Unit-Pharmacy Department, St Vincent’s University Hospital, Dublin, Ireland; 6 Aseptic Unit-Pharmacy Department, St Vincent’s Private Hospital, Dublin, Ireland; 7 Cancer Clinical Research Trust, St Vincent’s University Hospital, Dublin, Ireland; 8 Department of Pathology, St Vincent’s University Hospital, Dublin, Ireland; 9 Department of Surgery, St Vincent’s University Hospital, Dublin, Ireland; 10 Department of Radiation Oncology, St Luke’s Hospital, Dublin, Ireland

**Keywords:** Breast cancer, Targeted therapies

## Abstract

**Background:**

The optimal timing of (neo)adjuvant trastuzumab initiation with respect to chemotherapy and surgery remains undefined.

**Methods:**

Retrospective analysis of a large institutional database of HER2-positive patients who received anti-HER2 therapy. We included all Stage I to III patients treated with trastuzumab with a minimum follow up of 3 years. The date of first breast biopsy was recorded as initial diagnosis.

**Results:**

A total of 506 patients [adjuvant: 386 (76%)-neo-adjuvant: 120 (24%)] were included. The median time-to-first-trastuzumab (TFT) from diagnosis was 12 weeks (range 1.9–122.3). Median follow-up is 73.3 months (range 1.4–176.3). TFT was significantly shorter in the neo-adjuvant than in the adjuvant cohort (median: 4.4 vs. 14 weeks, *p* *<* 0.00001). Despite the neo-adjuvant cohort having significantly more node-positive patients (75 vs. 53%, *p* < 0.0001), DFS rate (neo-adjuvant: 12.5 vs. adjuvant: 18%, *p* = 0.094) was numerically superior in neo-adjuvant patients. A TFT ≤ 12 weeks was associated with significantly superior DFS and OS over TFT > 12 weeks. Early concomitant regimens were associated with superior DFS over delayed-concomitant and sequential regimens.

**Conclusions:**

Initiating trastuzumab more than 12 weeks from diagnosis has a negative impact on clinical outcome. Neo-adjuvant anti-HER2 therapy could be the optimal strategy to treat early stage HER2-positive breast cancer.

## Introduction

The incorporation of trastuzumab into adjuvant regimens for patients with HER2-positive breast cancer has revolutionised the outlook of this previously poor-prognosis sub-type. The results of four large randomised trials presented in 2005 all showed that chemotherapy plus trastuzumab produced superior survival compared to chemotherapy alone, rapidly defining a new standard of care.^1–3^

The regimens in these trials combined chemotherapy and trastuzumab in three different schedules. In the *early concomitant strategy*, trastuzumab was administered simultaneously with a taxane-containing chemotherapy from the start of treatment (e.g., one experimental arm of BCIRG006: TCH (docetaxel/carboplatin/trastuzumab)).^3^ In *delayed concomitant*, a trastuzumab-plus-taxane combination was delivered only after an initial phase of cyclophosphamide and doxorubicin therapy (as in B-31/NCCTG N9831 and one other experimental arm of the BCIRG006).^2,3^ Finally, the HERA trial looked at a purely *sequential strategy*, in which trastuzumab was administered as single agent following the completion of standard chemotherapy (with or without a taxane).^1^ Trastuzumab augmented the effect of chemotherapy in all of these studies and strategies. A smaller delayed concomitant trial, FinHER,^4,5^ in which trastuzumab was administered for nine weeks only, was also positive, although a small sequential trial, FNCLCC-PACS04, failed to show any benefit from the administration of trastuzumab after adjuvant chemotherapy.^6^ Systemic therapy can also be administered before definitive surgery to patients with breast cancer. This “neo-adjuvant” approach can downstage tumours, facilitating breast-conserving surgery, and in theory provides earlier systemic treatment of occult micro-metastases. A recent large meta-analysis of ten randomised clinical trials, conducted in the pre-trastuzumab era, has shown no significant difference between neo-adjuvant and adjuvant chemotherapy for distant recurrence, breast cancer mortality, or death from any cause.^7^

Neo-adjuvant trastuzumab for HER2-positive breast cancer has also been studied. In the NOAH trial, the incorporation of trastuzumab into an anthracycline-taxane neo-adjuvant regimen produced a higher rate of pathological complete response (pCR) and prolonged disease-free survival compared to the same pre-operative chemotherapy without trastuzumab.^8^

Attempts to improve on the results of treatment of HER2-positive breast cancer, have principally involved the incorporation of additional anti-HER2 drugs into existing regimens.^9,10^ There has been less focus on the possibility that optimised scheduling might improve outcomes. For example, the time interval between the initial diagnosis of breast cancer and the administration of the first dose of trastuzumab can vary hugely in clinical practice, ranging from a few weeks, in the case of neo-adjuvant early concomitant therapy, to over 6 months in the case of sequential adjuvant treatment. In two retrospective studies extreme delays in initiation of adjuvant trastuzumab, and delayed systemic therapy in general were both associated with inferior outcomes in HER2-positive disease.^11,12^ Neither study included neo-adjuvant treatments.

To date, no (neo)adjuvant trastuzumab-chemotherapy schedule has been proven to be superior to any other and no prospective or retrospective study has addressed neo-adjuvant trastuzumab-containing chemotherapy versus the same adjuvant therapy. In an attempt to address these issues we designed a study of all patients who were treated in our institution with anti-HER2 therapy for HER2-positive stages I-III breast cancer.

## Patients and methods

We conducted a retrospective analysis on a prospectively maintained database–called the *One Thousand HER2 Patients Project* – of all patients with HER2-positive breast cancer treated with trastuzumab at the Department of Medical Oncology of St Vincent’s University and St Vincent’s Private Hospitals in Dublin, Ireland. This database was established in 2010 to create a clinical resource for translational studies across all the different stages of HER2-positive breast cancer.

To be included in this study, patients must have Stage I to III invasive breast cancer which was HER2-positive in accordance to the international guidelines (i.e., ASCO/CAP guidelines) in use at the time of diagnosis, must have received at least one dose of trastuzumab-containing therapy, and have adequate follow up information. Also, full details on tumour biology (i.e., tumour grade, oestrogen [ER] and/or progesterone receptors [PgR]), as well as information on all pharmacological therapies administered (i.e., dates, schedules and doses of all cycles of treatments) had to be available for review. Patients diagnosed and followed up at our Institution but who received treatments, even only in part, elsewhere were excluded.

For all patients we recorded the dates of initial diagnosis of breast cancer (based on the first Pathology report showing invasive carcinoma), of definitive breast surgery, of the first and last administration of trastuzumab, and last follow up at our Institution or death. Most patients had a fine-needle aspiration (FNA) for cytological examination in case of abnormal axillary lymph nodes on imaging. Patients with unequivocal metastatic involvement of supraclavicular or internal mammary lymph nodes (Stage N3b and N3c)–confirmed by either FNA/core biopsy or PET-CT–and those who had inoperable locally advanced breast cancer were excluded from this study.

The clinical and pathological data of all patients deemed eligible for the study were individually reviewed and verified against the patients’ hospital medical records as the main source document.

In order to ensure that most patients had a minimum of three years of follow up, we included in the study only patients who received the first dose of trastuzumab before March 31st 2014. For those patients who relapsed after curative therapy, we recorded the date and the site of relapse (loco-regional or distant). The database was locked for outcome analyses on March 31st 2017. The Institutional Audit Committee of St Vincent’s University Hospital approved this study.

### End points and statistical considerations

#### Time to first trastuzumab

(TFT) was defined as the interval between the first diagnosis of HER2-positive invasive carcinoma of the breast (i.e., the diagnostic core biopsy) and the date of the first administration of trastuzumab, either in the neo-adjuvant or in the adjuvant setting.

#### Disease free survival

(DFS) was defined as the interval between the first administration of trastuzumab and the first evidence of relapsed invasive HER2-positive breast cancer, second primary breast cancer, second non-breast malignancy, death for any cause, whichever occurred first.

#### Overall survival

(OS) was calculated from the first administration of trastuzumab to the date of last follow up or date of death for any cause. Patients who died without evidence of disease recurrence were considered censored at the time of death.

Descriptive analysis was used to document the demographic and clinical data of the patients. Data were analysed using STATA 13.1. Comparisons between groups were performed by using the two-sided *χ*^2^ test, where appropriate for the detection of statistical significance. A *p* value <0.05 was considered statistically significant. Univariate and multivariate Cox regression analyses were used to determine independent prognostic predictors of recurrence and survival. Variables with a significant association in the univariate analysis were analysed with multivariate Cox regression for identifying independent factors. The Kaplan-Meier estimator method was applied to calculate survival, and the log-rank test was used for assessment of statistical significance.

## Results

### Patients’ characteristics and outcome

**Fig. 1:**
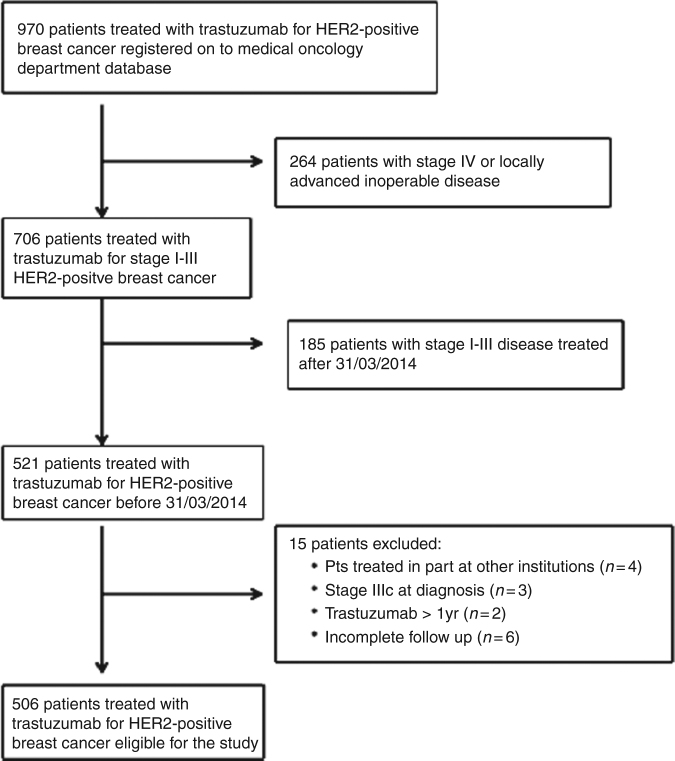
Flowchart of the study. The main source for this study was the *One Thousand HER2 Patient Project* database at St Vincent’s University Hospital, Dublin

Out of 970 patients with HER2-positive early stage breast cancer registered on the *One Thousand HER2 Patients Project* database as of December 31st 2016, we included in the study 506 patients treated between October 2001 and March 2014 who met all the pre-defined inclusion/exclusion criteria and form the overall population of the present study. Trastuzumab was administered as part of adjuvant therapy in 386 (76%) patients and of neo-adjuvant in 120 (24%). The study flowchart is shown in Fig. 1. Patients’ main characteristics are detailed in Table 1.

**Table 1:** Patients characteristics

Characteristic (%)	Total *(N* *=* *506)*	Neo-adjuvant *(N* *=* *120)*	Adjuvant *(N* *=* *386)*	*p-*value
Median age (range)	55 (26–85)	52 (26–74)	56 (26–85)	0.036
≤55	245	70 (58%)	175 (45%)	
>55	246	50 (42%)	196 (51%)	
Unknown	15	–	15 (4%)	
Tumour grade				
G1	10 (2%)	1 (1%)	9 (2%)	0.563
G2	153 (30%)	39 (32.5%)	114 (30%)	
G3	331 (65.5%)	76 (63%)	255 (66%)	
Unknown	12 (2.5%)	4 (3%)	8 (2%)	
Hormonal receptors status				
ER or PgR positive	321 (63%)	69 (57%)	252 (65%)	0.060
ER and PgR negative	166 (33 %)	47 (40%)	119 (31%)	
Unknown	19 (4%)	4 (3%)	15 (4%)	
Nodal status				
Positive	266 (52%)	86 (72%)	180 (47%)	<.0001
Negative	231 (46%)	29 (24%)	202 (52%)	
Unknown/Indeterminate	9 (2%)	5 (4%)	4 (1%)	
Time to first trastuzumab				
Median (range)	12 (1.9–122.3)	4.4 (1.9–17.9)	14 (6.7–122.3)	<.00001
Systemic therapy				
Early concomitant				
TCH	283 (56%)	84 (70%)	199 (52%)	<.00001
“TCH-like”	55 (10.9%)	27 (22%)	28 (7%)	
Other concomitant	15 (3%)	2 (2%)	13 (3%)	
Delayed concomitant				
AC-TH / FEC-TH	85 (16.7%)	7(6%)	78 (20%)	
Sequential trastuzumab	34 (6.7%)	0	34 (9%)	
Trastuzumab single-agent	34 (6.7%)	0	34 (9%)	
Pathological response (*N* = 120)				
Complete (pathCR)	–	52 (43%)	–	–
Non-complete		68 (57%)		

TCH (Docetaxel/Carboplatin/Trastuzumab), AC-TH (Doxorubicin/Cyclophosphamide-Docetaxel/Trastuzumab), FEC-TH (5-FU/Epirubicin/Cyclophosphamide-Docetaxel/Trastuzumab

Over a median follow-up of 73.3 months (range 1.4–176.3), in the overall population (*N* = 506) we observed 74 (15%) DFS events, accounting for a DFS rate of 85%, and 46 deaths (9%) accounting for an OS rate of 91%.

On univariate Cox regression analysis, patients’ age, hormone receptors status and tumour grading did not have a significant impact on DFS or OS. However, patients with positive axillary lymph nodes at initial diagnosis had significantly higher risk of recurrence (HR 2.40; 95%CI:1.44 to 4.02, *p* = 0.001) and death (HR 2.10; 95%CI:1.10 to 4.01, *p* = 0.024) compared to patients without lymph nodes involvement.

### Time to first trastuzumab (TFT) and outcome

**Fig. 2:**
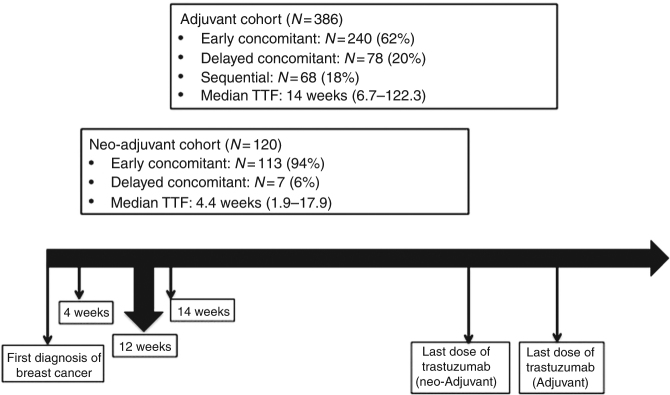
Timeline of trastuzumab initiation for the neo-adjuvant (median TFT: 4.4 weeks) and the adjuvant (median TFT: 14 weeks) cohorts in relation to the cut-off point of 12 weeks from the initial diagnostic biopsy. TFT = Time to first trastuzumab

TFT was calculated on 491 (97%) patients who had a recorded date of diagnostic breast biopsy before they underwent definitive surgery. The median TFT for the overall cohort was 12 weeks (range 1.9–122.3). When we dichotomised patients into initiating trastuzumab ≤12 weeks or >12 weeks from the diagnostic biopsy, patients age, ER and PgR status, tumour grading, and lymph node status did not differ significantly between the two groups. However, the proportion of patients who received the first dose of trastuzumab within 12 weeks from the diagnostic biopsy was significantly different between the neo-adjuvant and the adjuvant cohort: 95% in the neo-adjuvant cohort vs. 53% in the adjuvant cohort (mostly treated with TCH or “TCH-like” regimens). The timeline of trastuzumab initiation for the neo-adjuvant and neo-adjuvant cohorts is shown in Fig. 2.

**Fig. 3:**
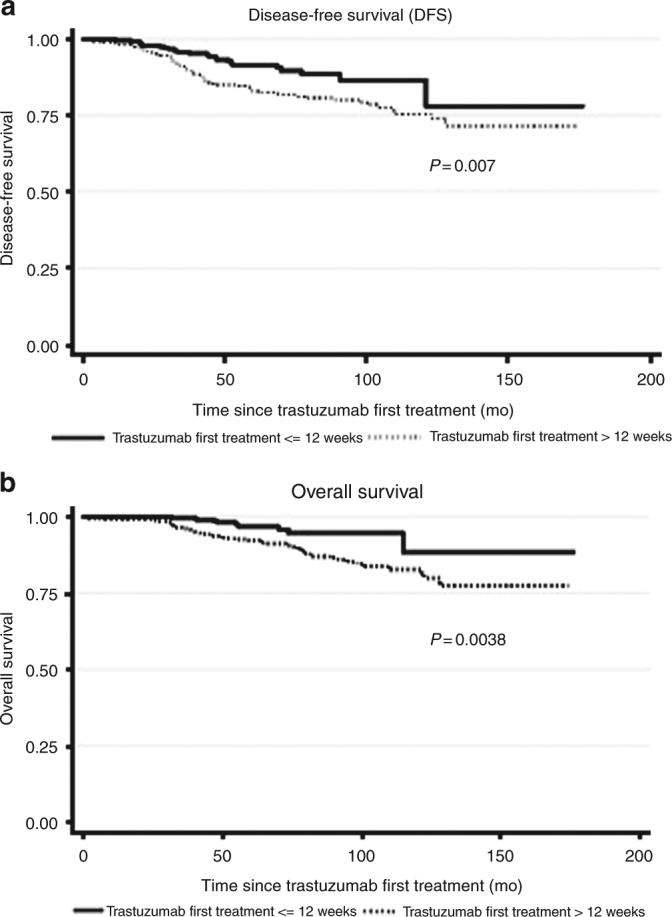
DFS (**a**) and OS (**b**) of patients with a TFT (time to first trastuzumab) ≤ 12 weeks (*N* = 247) compared with those with TFT > 12 weeks (*N* = 244)

Patients in the > 12 weeks group (*N* = 244) had a significantly higher risk of recurrence (HR 1.96; 95% CI:1.19 to 3.23, *p* = 0.008) and death (HR 2.84; 95% CI:1.36 to 5.96, *p* = 0.006) compared with patients in the ≤12 weeks group (*N* = 247). The 5-year DFS estimates were 91% [95% CI:86–94%] and 81% [95% CI:75–86%] in patients with TFT ≤12 weeks and in those with TFT > 12 weeks, respectively. The 5-year OS estimates were 97% [95% CI: 93–98%] and 91% [95% CI: 86–94%] in patients with TFT ≤ 12 weeks and in those with TFT > 12 weeks, respectively (Fig. 3).

### Adjuvant and Neo-adjuvant cohorts

The neo-adjuvant (*N* = 120) and the adjuvant (*N* = 386) cohorts did not differ significantly with regard to tumour grade, ER and PgR. However, patients in the neo-adjuvant cohort had a significantly higher rate of cytologically/histologically-positive lymph nodes than those in the adjuvant cohort (75 vs. 53%, *p* *<* 0.0001), indicating a more advanced disease stage at diagnosis. Also, as expected, patients in the neo-adjuvant cohort received the first dose of trastuzumab significantly earlier than those treated adjuvantly: TFT was highly significantly shorter in the neo-adjuvant compared to the adjuvant cohort (median TFT: 4.4 vs. 14 weeks, *p* < 0.00001). Despite the significantly higher rate of lymph node positivity in the neo-adjuvant cohort, DFS rates were numerically superior for the patients treated pre-operatively (neo-adjuvant: 12.5% vs. adjuvant: 18%, *p* = 0.094). OS was identical in the two cohorts. We observed a significant level of interaction between the nodal status at diagnosis (positive vs. negative) and the setting of trastuzumab-based therapy (neoadjuvant vs. adjuvant) in DFS (*p* = 0.0066) but not OS (*p* = 0.1175).

It’s been our long-standing Institutional policy to initiate adjuvant chemotherapy within 8 weeks from the date of the last surgical procedure. However, the date of the first administration of trastuzumab varied, depending on the specific therapeutic regime chosen. When we analysed the impact on patients’ outcome of the timing of first trastuzumab exclusively in the adjuvant cohort (*N* = 386), we observed that DFS (94 vs. 85%, *p* = 0.029) and OS (98 vs. 92%, *p* = 0.0047) were significantly superior in patients who started trastuzumab ≤ 8 weeks compared to >8 weeks, respectively (median FU 84 months, range 1–176).

### Trastuzumab-containing strategies and outcome

**Fig. 4:**
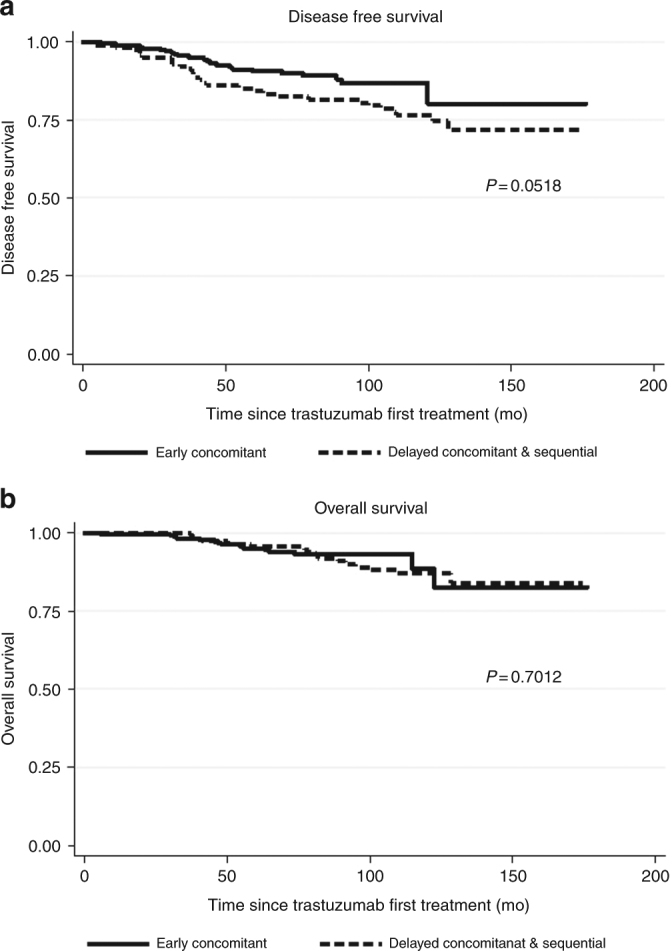
DFS (**a**) and OS (**b**) of patients treated with *early concomitant* regimens (e.g., TCH and “TCH-like”) compared with those treated with *delayed concomitant* (e.g., AC-TH) and *sequential* regimens. TCH (Docetaxel/Carboplatin/Trastuzumab), AC-TH (Doxorubicin/Cyclophosphamide-Docetaxel/Trastuzumab)

Approximately 70% of patients registered in our database were treated according to an *early concomitant strategy*. When we analysed the impact of the different trastuzumab-containing regimens on patients outcome, we found that patients treated with a *delayed concomitant* (mostly with AC-TH regime) and a *sequential strategy* (single-agent trastuzumab as in the HERA trial) had an increased risk of disease relapse compared to patients treated with an early concomitant strategy (mostly TCH and “TCH-like” regimens) (DFS HR 1.86 (95% CI:1.11–3.09), *p* = 0.017). The difference in OS was not statistically significant, most likely due to the small number of events in the *early concomitant* group [OS HR 1.18 (0.59–2.34), *p* = 0.629] (Fig. 4).

### Multivariate analyses

We performed a multivariate analysis based on significant clinical factors from the univariate analysis. In the multivariate model, positive lymph nodes (DFS HR 2.30 95% CI:1.37–3.84 *p* = 0.001; OS HR 2.02 95% CI:1.05–3.85 *p* = 0.033), TFT (≤12 weeks vs. >12 weeks), (DFS HR 1.85 95% CI:1.12–3.05 *p* = 0.016, OS HR 2.54 95% CI:1.21–5.35 *p* = 0.014) and patients age (dichotomised ≤55 vs. >55 years) (OS only HR 2.07 95% CI:1.13–3.80 *p* = 0.018) remained significant and independent variables associated with an increased risk of relapse and death.

## Discussion

In an attempt to improve adjuvant therapy of HER2-positive breast cancer, the incorporation of additional anti-HER2 agents into existing regimens has been studied. In the Neo-ALTTO^9^ and ALTTO^13^ trials the anti-HER2 tyrosine kinase inhibitor lapatinib failed to improve disease-free or overall survival. Neratinib, a newer TKI, has however produced an absolute 2.5% increase in relapse-free survival, but no difference in overall survival when used following completion of 12 months of adjuvant trastuzumab.^14^

The results of the APHINITY trial showed that while the addition of pertuzumab to chemotherapy and trastuzumab produced a statistically significant improvement in invasive disease-free survival for patients with HER2-positive disease, the difference in absolute terms was so small as to be of dubious clinical significance. In addition, there was no improvement in survival.^13,15^ Could optimal scheduling of existing trastuzumab and chemotherapy produce clinical benefits?

Very limited in-trial comparisons of different adjuvant chemotherapy-trastuzumab schedules are available, and no prospective trials have addressed the more fundamental comparison of adjuvant versus neo-adjuvant therapy. Two studies, BCIRG 006 and NCCTG N9831, did address scheduling, but in only one of these, the NCCTG, was the effect of schedule isolated. In two arms of NCCTG, identical chemotherapy and trastuzumab were administered, with trastuzumab given either following completion of all chemotherapy or simultaneously with paclitaxel as a component of a sequential anthracycline and taxane-based chemotherapy. While both schedules produced statistically significant improvements in survival compared to identical control chemotherapy without trastuzumab, the combination regimen was numerically superior.^2^

In BCIRG 006 the two trastuzumab arms differed both in the timing of trastuzumab and in the chemotherapy agents used. Both were significantly superior to chemotherapy alone, with no statistically significant differences between early concomitant and late concomitant trastuzumab arms, despite the late arm containing anthracycline, having more cycles of chemotherapy, and causing more toxicity.^3^

Our data might suggest an explanation why these studies did not produce significant advantages for an early schedule of trastuzumab. The impact of difference in time to trastuzumab may be blunted by the fact that all adjuvant regimens should be considered by definition as *delayed* regimens if compared to *early concomitant* given neo-adjuvantly. Two retrospective studies have shown that delaying the initiation of postoperative adjuvant therapy is associated with worse survival for patients with HER2-positive disease.^11,12^ Investigators at MD Anderson^11^ analysed a cohort of HER2-positive patients treated with chemotherapy with or without trastuzumab and found that the detrimental effect of delayed systemic therapy on OS (but not on RFS) was observed only in the trastuzumab-treated group (*N* = 591). Patients who started adjuvant treatment ≥61 days had inferior outcomes compared with those who started treatment ≤30 days after surgery. This study did not address the impact of different adjuvant regimens, the timing of the institution of trastuzumab, nor the timing from time of diagnosis. Importantly, it did not include neoadjuvant regimens.

In our study, the timing of trastuzumab was a significant determinant of outcome, with a highly significant difference in DFS and OS for those receiving trastuzumab within and those beyond 12 weeks. Furthermore, the initiation of trastuzumab longer than 8 weeks from the date of surgery in the adjuvant cohort had a detrimental effect on both DFS and OS, thus strengthening the observation that TFT is a clinically meaningful factor and that anti-HER2 therapy should be initiated as soon as possible in the patients’ treatment pathway.

While the potential limitations of retrospective analysis are acknowledged, the adjuvant versus neo-adjuvant data in our study should at least provide reassurance that neo-adjuvant therapy is not at all likely to produce inferior survival outcomes compared to post-operative adjuvant therapy. It must be emphasised that the case selection used in the adjuvant-versus-neo-adjuvant decision should have biased the results against the neo-adjuvant therapy cohort, which had a significantly higher proportion of patients with positive nodal involvement at diagnosis. However, the strengths of this study include its being based on an institutional prospectively maintained database, the completeness of clinical and pathological data for all patients, and the long follow up (median >6 years). Thus, although a significantly higher proportion of patients in the neo-adjuvant cohort of our study had positive lymph nodes, and hence a worse prognosis, the DFS and OS they achieved were identical to the lower risk adjuvant group.

Several biological hypotheses can be made to try to explain the findings of our study. First of all, there is documented pre-clinical synergy between trastuzumab and taxanes.^16^ Also, in experimental animals the interval between tumour removal and administration of cytotoxic chemotherapy was critical and the most effective control of metastases could be obtained when chemotherapy was administered before primary tumour removal, thus providing a biological rationale for the administration of pre-operative systemic therapy.^17^

Targeting HER2 from the first day of cytotoxic chemotherapy can maximise the benefits of systemic therapy and increase the activity on occult micrometastatic disease. Neo-adjuvant trastuzumab-based chemotherapy might also optimise the interactions between the tumour and the host’s immune system, especially CD8+ T-cells among the others,^18^ and also enhance trastuzumab-induced ADCC (antibody-dependent cellular cytotoxicity).^19^

As health systems face the prospect of the significant financial toxicity of buying additional, very expensive anti-HER2 agents, the possibility that a cost neutral re-formatting of existing treatments might also produce similar or even higher degrees of improvement is of major potential economic significance. Given the current industrial dominance of the clinical trials systems, it seems highly unlikely that a prospective and adequately powered comparison of adjuvant versus neo-adjuvant use of drugs, which are in the later stages of their patent protection, will ever occur.

In conclusion, our large, mature retrospective study confirms and extends prior reports that delaying anti-HER2 therapy has a negative clinical impact. Our study has three novel observations. Firstly, it is not only extreme delays (i.e.,>6 months) that are deleterious. Minor delays, such as those that are frequently encountered in the real world, also have a negative impact. The initiation of trastuzumab more than 12 weeks from initial diagnosis, as well as more than 8 weeks from surgery has a significantly negative impact and should be avoided, especially in node-positive patients. Secondly, the timing of the specific anti-HER2 therapy, and not the time to chemotherapy is the most important schedule variable.

Finally, and perhaps most controversially, based on the above observations we suggest that the neo-adjuvant should be considered as the preferable approach over adjuvant anti-HER2 therapy. This is now our institutional standard for all newly diagnosed HER2-positive early-stage invasive breast cancers. Other institutions with large databases should be encouraged to perform similar analyses.

## Availability of data and materials

The full data supporting the results of this study can be found in the Medical Oncology Clinical Research Unit of St Vincent’s University Hospital, Elm Park, Dublin, Republic of Ireland.
